# From Resilience to Resistance: Rethinking Faculty Well‐Being as a Moral and Political Problem in Nursing Education—Toward a Humane Ethics of Academic Care

**DOI:** 10.1111/nin.70092

**Published:** 2026-02-23

**Authors:** Suha Ballout, Samira Hamadeh

**Affiliations:** ^1^ Department of Biobehavioral Nursing & Health Informatics University of Washington School of Nursing Seattle Washington USA; ^2^ Institute of Health and Wellbeing Federation University Australia Berwick Australia

**Keywords:** academic care, decolonial practice, faculty well‐being, institutional ethics, moral injury, moral resilience, nursing education, tempered radicalism

## Abstract

This paper explores faculty well‐being in nursing education as a moral and political issue, emphasizing a humane ethics of academic care that confronts institutional harm, moral distress, and inequality. Despite nursing's commitments to compassion, equity, and justice, many educators face excessive workloads, racial exclusion, and moral conflicts, leading to burnout and moral injury. Current wellness approaches individualize distress and hide institutional responsibility. Drawing on critical, decolonial, and ethical traditions, the paper challenges resilience‐based discourses, framing faculty well‐being as a collective moral obligation rooted in governance and power. It synthesizes decolonial scholarship, moral resilience, transformational leadership, and human rights, grounded in ethical principles and ESG standards. Using examples from faculty development and institutional practice, it introduces the Becoming HUMANE Framework as a lens, not a model, to understand healing, rights, resilience, accountability, belonging, and empowerment as essential to ethical academic environments. Nurse educators are positioned as tempered radicals whose reflective resistance turns moral distress into collective agency and accountability. Reframing well‐being as a moral and political issue reveals the limits of individual resilience and advocates for humane academic systems. Nursing education must address institutional conditions affecting educator well‐being to uphold its moral commitments.

## 1 Introduction

Nursing is a science and a moral commitment, a profession rooted in compassion, justice, and the protection of human dignity (Flaubert et al. 2021; ICN 2025; National Academies of Sciences, Engineering, and Medicine [NASEM] 2021). Because it combines intellect, empathy, and advocacy, nursing education has a dual responsibility to cultivate clinical competence, foster critical awareness, and prepare practitioners to confront suffering, inequity, and ethical complexity with humility and courage (De Sousa et al. 2024; Flaubert et al. 2021; Giordano et al. 2024; Liimatainen 2001; Roy et al. 2022). Yet, within the very institutions that promote these ideals, a quiet but significant contradiction persists. Those who teach compassion, equity, and care often work within systems that foster exclusion, hierarchy, and moral harm (Ballout et al. 2025; Bell 2021; Coleman 2020; Iheduru‐Anderson and Waite 2022, 2024; Zappas et al. 2021). This predicament creates a moral and structural paradox that is professional and political, reflecting how academic nursing institutions distribute power, recognition, and risk in ways that can undermine the ethical commitments they publicly espouse (Ballout et al. 2025; Bell 2021; Coleman 2020; Hammoudi Halat et al. 2023; Iheduru‐Anderson and Waite 2022, 2024; Toescher et al. 2020; Zappas et al. 2021). Systematic reviews indicate that most nursing faculty experience moderate to high burnout, with exhaustion, role strain, and moral distress linked to heavy workloads, lack of recognition, and systemic inequities (Ballout 2025b; Hosseini et al. 2022; Shirey 2006; Singh et al. 2020; Watson 2023). While burnout is often described as an individual outcome, it can also signal institutionally produced moral injury when educators are repeatedly constrained from acting in accordance with their professional values. At the same time, persistent faculty shortages have created a critical bottleneck in nursing education; in 2022 alone, over 78,000 qualified applicants were denied admission to US programs due to insufficient faculty and institutional resources, resulting in downstream effects on workforce development and clinical training capacity (American Association of Colleges of Nursing [AACN] 2021; Boamah et al. 2023; Metersky et al. 2025). These conditions threaten the sustainability of the nursing workforce and the moral integrity of the profession: when educators who teach healing do so from environments that compromise their own well‐being, the contradiction becomes existential (Flaubert et al. 2021; Hughes and Rushton 2022).

Globally, nursing organizations explicitly frame this situation as urgent. The International Council of Nurses projections of a nearly 6‐million nurse shortfall, the Global Strategic Directions for Nursing and Midwifery 2021–2025, and the National Plan for Health Workforce Well‐Being all call for safe, empowering, and equitable work environments and position clinician and educator well‐being as a shared organizational and policy responsibility rather than an individual trait (Flaubert et al. 2021; Hammoudi Halat et al. 2023; ICN 2025; National Academies of Sciences et al. 2021; WHO 2021). Despite these mandates, wellness initiatives in nursing academia continue to prioritize individual coping, mindfulness, self‐care, and time management workshops while essentially leaving unaddressed the structural drivers of distress (Luckett 2020; Ruth‐Sahd and Grim 2021; Stephens and Clark 2024; Waugh et al. 2025). These dynamics are intensified for faculty from minoritized backgrounds, who shoulder disproportionate emotional labor, racialized scrutiny, and “cultural taxation,” reflecting colonial continuities in nursing education that maintain epistemic dominance under the language of diversity and social justice (Akintade et al. 2025; Al‐Chami et al. 2024; Ballout et al. 2025; Bell 2021; Garland and Batty 2021; Iheduru‐Anderson 2025a, 2025b; Iheduru‐Anderson and Waite 2022; Slemon et al. 2024; Valderama‐Wallace and Apesoa‐Varano 2020; Zappas et al. 2021). This state results in moral fatigue and disengagement rather than resilience (Hammoudi Halat et al. 2023; Hosseini et al. 2022; Toescher et al. 2020).

Nursing education is increasingly shaped by the political economy of higher education, where market‐oriented logics, performance metrics, and revenue dependence intersect with the moral labor of care work (Alexander 2025; Evans 2007; Pope‐Ruark 2024; Verna and D'Andreamatteo 2025). Schools of nursing occupy a unique and precarious position at the nexus of academia, the healthcare industry, and public accountability, while often operating under conditions of chronic underfunding, escalating enrollment demands, and shrinking institutional investment (Boamah et al. 2023; Flaubert et al. 2021; ICN 2025, 2; National Academies of Sciences et al. 2021). Program closures, hiring freezes, consolidation of departments, and reductions in faculty lines are not merely technical or budgetary adjustments; they are moral decisions with direct consequences for educational capacity, workforce sustainability, and the ethical climate of academic life (Flaubert et al. 2021; Hughes and Rushton 2022; National Academies of Sciences et al. 2021; WHO 2021). Importantly, these conditions are not abstract. Colleagues and administrators make situated decisions about whether to authorize searches, sustain programs, or redistribute resources that materially shape which labor is valued, which scholarship is supported, and which voices are retained (Bewer et al. 2025; Iheduru‐Anderson 2025a, 2025b; Owen et al. 2018). For faculty, particularly those already navigating structural marginalization, repeated exposure to disinvestment and institutional silence in the face of advocacy constitutes a distinct form of moral distress: the experience of recognizing harm, naming alternatives, and yet remaining structurally constrained from preventing ethically troubling outcomes (Hughes and Rushton 2022; Rushton et al. 2021; Stephens and Layne 2023; Toescher et al. 2020).

This paper adopts a critical conceptual approach to examine faculty well‐being in nursing education not as an individual problem of resilience, but as a moral and political issue embedded in institutional culture, governance, and power relations. Drawing on decolonial scholarship, moral resilience and moral injury literature, and human rights–based perspectives, we interrogate the limits of prevailing wellness discourses and reframe academic care (i.e., the ethical responsibility institutions hold toward those who teach care) as an institutional ethical obligation. Within this analysis, the HUMANE Framework is advanced as a conceptual lens rather than a prescriptive framework to understand how Healing, Upholding Rights, Moral Resilience, Accountability, Nurturing Belonging, and Empowerment operate as interdependent conditions for humane academic environments. This paper aims to (1) theorize the moral paradox shaping contemporary nursing education; (2) critically examine how institutional practices produce moral injury among faculty; (3) articulate a humane ethics of academic care; and (4) consider how faculty resistance and collective agency can reorient nursing education toward moral integrity and justice. In doing so, this analysis calls for a shift from coping to collective courage, from endurance to empowerment, and from isolated wellness initiatives to systemic healing in nursing education (Flaubert et al. 2021; National Academies of Sciences et al. 2021; WHO 2021).

## 2 Background

The well‐being of nursing faculty, moral and physical, has become a significant concern and a key factor in the profession's ability to educate, support, and motivate a workforce that can promote health equity (Boamah et al. 2023; Flaubert et al. 2021; Hammoudi Halat et al. 2023; Hosseini et al. 2022; Watson 2023). The State of the World's Nursing 2025 report warns of a projected 6 million nursing shortage, citing inadequate educational infrastructure and faculty attrition as the leading causes (ICN 2025). The Global Strategic Directions for Nursing and Midwifery 2021–2025 emphasizes the need for safe, empowering, and equitable workplaces for faculty, as these are vital to maintaining the workforce (WHO 2021). The National Academy of Medicine (National Academies of Sciences et al. 2021) also considers clinician and educator well‐being a moral and organizational duty, one that calls for cultural and structural reforms (Hughes and Rushton 2022; Luckett 2020; Singh et al. 2020). Therefore, faculty health is not just a private issue but a systems‐level ethical obligation that is crucial to upholding nursing's moral purpose and sustaining a globally resilient workforce (Flaubert et al. 2021; ICN 2025; WHO 2021).

### 2.1 Faculty Burnout, Moral Distress, and Institutional Pressures

Escalating workloads, dwindling resources, and the emotional strain of caregiving in classroom and clinical environments left many educators exhausted (Hamadeh et al. 2025; Hammoudi Halat et al. 2023; Singh et al. 2020). Systematic reviews indicate that burnout affects over 70% of nurse faculty, driven by work overload, student incivility, and insufficient administrative support (Hosseini et al. 2022; Singh et al. 2020; Watson 2023). Faculty distress is further intensified by a pervasive sense of urgency generated by external mandates, accreditation cycles, continuous assessment requirements, and performance management systems operating on accelerated timelines (Alexander 2025; Owen et al. 2018; Pope‐Ruark 2024). Faculty are routinely expected to respond to new initiatives, data requests, curricular revisions, and compliance demands without corresponding release time, staffing, or workload recalibration (Flaubert et al. 2021; National Academies of Sciences et al. 2021). These mechanisms of evaluation produce a chronic state of temporal scarcity in which the ethical, relational, and reflective dimensions of academic work are crowded out by perpetual, deadline‐driven labor (Hammoudi Halat et al. 2023; Hata et al. 2022; Verna and D'Andreamatteo 2025). Such conditions deepen moral distress when educators recognize that meaningful teaching, mentorship, and equity work require time and care that institutions rhetorically value but structurally fail to protect (Hughes and Rushton 2022; Rushton et al. 2021; Stephens and Layne 2023). Qualitative research indicates that moral distress arises from conflicts between faculty professional values and institutional constraints, especially when decisions compromise student fairness or patient safety (Al‐Rjoub et al. 2022; Iheduru‐Anderson 2025a, 2025b; Toescher et al. 2020). Faculty describe handling “emotional labor without reprieve,” a situation that erodes psychological safety and collegial trust (Beard and Johnson 2024; L. Butler and Lyman 2025). The accumulated stress contributes to the experience of moral injury, creating a profound disconnect between nursing's ethical ideals and the realities of academic life (Hughes and Rushton 2022; Rushton et al. 2021; Stephens and Layne 2023). Unlike burnout, moral injury implicates institutional arrangements that constrain ethical agency rather than individual coping capacity.

### 2.2 Structural and Cultural Roots

Importantly, these political–economic pressures are enacted not only through budgetary decisions but also through temporal governance: the normalization of constant urgency, rapid turnaround expectations, and unfunded mandates that reorganize academic labor around speed rather than care (Alexander 2025; Owen et al. 2018; Pope‐Ruark 2024). Urgency itself becomes a mechanism of control that disciplines faculty into compliance while eroding the conditions necessary for ethical, relational, and justice‐oriented work (Hammoudi Halat et al. 2023; Hughes and Rushton 2022; Verna and D'Andreamatteo 2025). Beyond individual exhaustion, entrenched cultural legacies of hierarchy, gendered norms, and racial inequity persist. Academic nursing has long reflected colonial, patriarchal, and racialized structures that reproduce exclusion even within diversity discourses (Bell 2021; Coleman 2020; Iheduru‐Anderson and Waite 2024). Ballout et al. (2025) described these as colonial continuities, systems that promote social justice but maintain epistemic dominance and silence. Waite and Nardi (2019) further trace these hierarchies to nursing's historical subservience within medicine, where obedience was equated with professionalism (Waite and Nardi 2019). Such conditions marginalize voices advocating for transformation and normalize endurance over empowerment. In this context, resistance, whether through refusal, critique, or collective action, often becomes a necessary ethical response rather than a sign of disengagement. Faculty who resist often face subtle forms of retaliation or isolation, leading to climates of moral fear and disengagement (C. M. Clark and Springer 2010; Slemon et al. 2024; Small et al. 2024).

### 2.3 Structural and Cultural Roots

Moral distress in nursing education is produced not only by interpersonal dynamics and cultural norms but also by governance structures and political–economic choices (Alexander 2025; Owen et al. 2018; Pope‐Ruark 2024; Verna and D'Andreamatteo 2025). Decisions to sunset programs, delay or cancel faculty searches, increase teaching loads, or prioritize revenue‐generating initiatives over educational infrastructure reflect institutional value hierarchies that often conflict with nursing's stated commitments to equity, access, and social responsibility (Flaubert et al. 2021; Hughes and Rushton 2022; National Academies of Sciences et al. 2021; WHO 2021). When faculty engage in cross‐departmental or cross‐college advocacy to resist these patterns, organizing, submitting proposals, presenting data, and building coalitions, yet encounter institutional inaction or continued cuts, the result is not simply frustration (Bewer et al. 2025; Iheduru‐Anderson 2025a, 2025b; Small et al. 2024). It is a cumulative moral injury born of witnessing foreseeable harm, exercising ethical agency, and encountering systemic indifference (Rushton et al. 2021; Stephens and Layne 2023; Toescher et al. 2020). This form of moral distress is collective, structural, and political, underscoring the need to move beyond individualized accounts of burnout (Hammoudi Halat et al. 2023; Hosseini et al. 2022; Singh et al. 2020).

### 2.4 Psychological Safety and Institutional Culture

Recent studies highlight that psychological safety is crucial for learning and well‐being (Beard and Johnson 2024; L. Butler and Lyman 2025; Dale‐Tam et al. 2021). Faculty and students flourish when they can share concerns, make mistakes, and innovate without fear of retribution (Beard and Johnson 2024; L. Butler and Lyman 2025; Edmondson 2019). However, hierarchical leadership structures, incivility, and competitive or punitive cultures frequently erode this safety, limiting open dialogue and relational trust (Ackerman‐Barger et al. 2021; J. Clark 2025). Mrayyan (2024) found that authentic and humble leadership predicted higher psychological safety in academic teams, whereas a lack of empathy and transparency was associated with burnout (Mrayyan and Al‐Rjoub 2024). Stephens and Clark (2024) argue that civility and relational resilience are closely tied to moral integrity, necessitating organizational accountability that extends beyond interpersonal responsibility (Stephens and Clark 2024).

### 2.5 The Limits of Individualized “Resilience” Models

Despite evidence of systemic causes, institutional responses focused on individual coping strategies, such as mindfulness sessions, time‐management training, and wellness apps, rather than structural reform (Hata et al. 2022; Stephens and Clark 2024). Such programs may inadvertently individualize systemic distress by shifting the responsibility for adaptation onto those most affected by structural inequities, thereby obscuring the need for broader institutional and societal transformation, while also recognizing that structural change alone is insufficient without parallel investment in individual and relational capacities that support faculty well‐being and moral agency (Hughes and Rushton 2022; National Academies of Sciences et al. 2021; Rushton et al. 2021). At the same time, a growing body of literature affirms that resilience is an important protective resource that can buffer stress, support adaptive coping, and sustain professional functioning in the face of adversity (Abdelrahman et al. 2025; Ali and Shaban 2025; Rajamohan et al. 2025). Individual capacities such as emotional regulation, reflective practice, and moral grounding play a meaningful role in how educators navigate complex and demanding environments. However, evidence consistently demonstrates that resilience is neither a fixed trait nor a substitute for working conditions alone. Rather, resilience is best understood as a dynamic capacity that is cultivated, supported, or eroded by organizational context (Hughes and Rushton 2022; Rushton et al. 2021; National Academies of Sciences et al. 2021). Investments in individual capability development are, therefore, necessary but insufficient in isolation. When resilience initiatives are decoupled from structural reform, they risk shifting responsibility for systemic harm onto individuals while leaving the conditions that produce distress intact (Stephens and Clark 2024; Hammoudi Halat et al. 2023; Hata et al. 2022). Accordingly, individual‐level resilience and structural change must be pursued concurrently: strengthening individual capacities while simultaneously transforming governance, workload structures, leadership practices, and cultures of accountability. Within this framing, resilience functions not as the endurance of harmful systems but as a capacity activated and sustained through empowerment, psychological safety, and institutional justice. Ali and Shaban (2025) and Rajamohan et al. (2025) found that resilience thrives only in environments of empowerment and supportive leadership (Ali and Shaban 2025; Rajamohan et al. 2025). Abbasi et al. (2019) also demonstrated that moral empowerment interventions reduce moral distress, confirming that empowerment, not stoicism, is the antidote to burnout (Abbasi et al. 2019). True moral resilience thus relies on organizational justice, transparent governance, and cultures that foster compassion and respect for human rights (Hughes and Rushton 2022; ICN 2021, 2025; WHO 2021; Rushton et al. 2021).

### 2.6 Intersectionality, Representation, and Moral Burden

Faculty from underrepresented backgrounds bear additional burdens of racialized scrutiny, invisibility, and “cultural taxation” (Akintade et al. 2025; Iheduru‐Anderson 2025a), in addition to structural discrimination in hiring, promotion, and leadership pathways, which increase stress and attrition (Bewer et al. 2025). Faculty of color navigate mentorship deserts and racial microaggressions while being expected to lead diversity initiatives, which can lead to moral exhaustion (Iheduru‐Anderson 2025b). The importance of antiracist and decolonial praxis within curricula and governance is essential to institutional integrity (Coleman 2020); therefore, nurse leaders call for decolonizing nursing education through critical pedagogy that reclaims nursing's emancipatory and global roots (Ballout et al. 2025; Iheduru‐Anderson and Waite 2024). Despite their role in leading diversity efforts, they often lack sufficient mentorship and support, leading to moral exhaustion.

Moral resilience refers to the capacity of individuals and collectives to sustain or restore moral integrity in response to moral adversity, ethical uncertainty, and institutional constraint (Rushton et al. 2021; Hughes and Rushton 2022). Moral injury denotes the lasting psychological, emotional, and relational harm that occurs when individuals are repeatedly exposed to situations in which their deeply held values are compromised or violated (Rushton et al. 2021; Stephens and Layne 2023). Moral courage is understood as the willingness to speak up and act in accordance with ethical commitments despite personal or professional risk (Hughes and Rushton 2022). Moral imagination refers to the capacity to envision just and humane alternatives to existing practices and structures (Stephens and Layne 2023). Moral integrity describes the coherence between one's espoused values and enacted practices across individual and institutional contexts (Rushton et al. 2021; Hughes and Rushton 2022). We use these concepts collectively to describe the moral terrain nurse educators navigate.

### 2.7 Leadership, Empowerment, and Moral Integrity

Moral integrity, as used in this paper, refers to the coherence between individuals' and institutions' espoused ethical values and their enacted practices, particularly in relation to dignity, equity, and responsibility for preventing harm. It reflects the capacity to recognize ethical obligations, to act in accordance with professional and moral commitments, and to sustain this alignment despite competing demands or structural constraints (Hughes and Rushton 2022; Rushton et al. 2021). At the institutional level, moral integrity is expressed through governance structures, leadership behaviors, and accountability mechanisms that consistently translate stated commitments to compassion, justice, and well‐being into concrete policies and everyday practices (National Academies of Sciences et al. 2021; Owen et al. 2018). Leadership style shapes faculty well‐being: transformational and empowering leadership are associated with higher job satisfaction and lower burnout (Bass and Riggio 2006; Cummings et al. 2008; Sarmiento et al. 2004). Mrayyan and Al‐Rjoub (2024) demonstrated that humble and authentic leadership fosters creativity and psychological safety, whereas authoritarian cultures are associated with disengagement (Mrayyan and Al‐Rjoub 2024). Empowerment theory (Kanter 1977) and related evidence (Baker et al. 2011; Greco et al. 2006) underscore that access to resources, information, and support fosters professional vitality and moral agency. Conversely, disempowerment produces ethical erosion and resignation. The absence of participatory governance, transparency, and shared accountability undermines morale, as well as educational quality and justice outcomes (National Academies of Sciences et al. 2021^2021^; Owen et al. 2018).

### 2.8 From Endurance to Structural Healing

Reclaiming moral integrity requires transforming institutions from cultures of endurance to systems of healing. Moral integrity, the congruence between espoused values and enacted practice, anchors professional identity and ethical trust (Rushton et al. 2021, 2022). When compassion and justice are absent from the workplace, educators experience moral injury and a loss of professional meaning (Hughes and Rushton 2022). Transformational leadership, trauma‐informed pedagogy, and participatory governance provide pathways toward renewal (Cummings et al. 2008; Evans‐Agnew et al. 2025). Human rights frameworks (Biluan 2025; Yamin 2015) and Environmental, Social, and Governance (ESG) principles (Sueyoshi and Goto 2025; Verna and D'Andreamatteo 2025) affirm that ethical institutions safeguard the dignity, safety, and development of their workforce. Restoring moral integrity requires aligning values with practice, addressing moral injury, and embedding ethical, inclusive leadership. The *HUMANE* Framework responds to the Future of Nursing 2020–2030 call to foster equity and clinician well‐being (Flaubert et al. 2021) by reframing faculty well‐being as a shared ethical and structural responsibility, rather than an individual burden.

## 3 Theoretical Foundations

The HUMANE Framework is grounded in intersecting critical, ethical, and organizational theories that illuminate how institutional power, governance, and culture shape faculty moral agency and well‐being. These perspectives show how systems of domination and institutional inertia undermine the health and moral agency of nurse educators and how re‐centering healing, justice, and empowerment can restore moral integrity to nursing education (Ballout et al. 2025; ICN 2025; Iheduru‐Anderson and Waite 2024).

Oppression and coloniality are deeply rooted in the historical foundations of nursing education. Drawing on Freire's critical pedagogy, oppression is not merely an interpersonal problem, but a systemic process of dehumanization that perpetuates inequality by restricting critical consciousness and calls for conscientização, a praxis of reflection and action through which individuals and communities reclaim agency (Freire 1978). Similarly, Fanon exposed the psychic and structural violence of colonial domination, where colonized people internalize feelings of inferiority and institutions uphold imperial hierarchies (Burke 1976). Waite and Nardi (2019) expand these ideas to nursing, showing how colonialism shaped the profession's hierarchies, gendered obedience, and moral conformity (Waite and Nardi 2019). They describe “nursing colonialism” as a system that favors compliance over critical engagement. Ballout (2025) builds on this critique by framing decolonizing nursing education as a process of reclaiming diverse ways of knowing, dismantling knowledge hierarchies, and viewing healing as both personal and structural transformation (Ballout et al. 2025). Likewise, Iheduru‐Anderson (2023) sheds light on the lived experiences of Black nurse educators who navigate racialized surveillance, tokenism, and expectations to show gratitude within predominantly white institutions (Iheduru‐Anderson 2025a, 2025b). These experiences undermine psychological safety and collective efficacy. Faculty well‐being is closely tied to structural power: chronic stress, moral distress, and burnout are not signs of personal weakness but effects of institutionalized inequality and epistemic exclusion (Hosseini et al. 2022; Singh et al. 2020). The HUMANE Framework's Healing, Upholding Rights, and Empowerment dimensions directly respond to these issues. Healing involves recognizing institutional harm, creating spaces for repair, and adopting trauma‐informed practices that validate faculty experiences. Upholding Rights emphasizes the recognition of dignity, equity, and justice as essential professional commitments. Empowerment requires redistributing voice, resources, and authority within academia, shifting from paternalistic governance toward shared leadership and collective agency. Together, these elements reflect what Freire and Fanon envisioned: liberation through awareness, solidarity, and transformation (Burke 1976; Freire 1978).

Healing institutional harm also requires leadership that demonstrates moral courage and empathy. Transformational leadership theory highlights four leadership styles: inspirational motivation, intellectual stimulation, individualized consideration, and idealized influence (Bass and Riggio 2006; Boamah 2022; Cummings et al. 2008). Such leaders inspire purpose, exemplify integrity, and foster a sense of belonging. When applied to nursing education, transformational leadership redefines faculty and deans as co‐creators of compassionate systems that emphasize relational ethics and shared responsibility (Boamah 2022). Supporting this thought, Edmondson (2019) describes psychological safety as the belief that one can speak up, ask questions, and challenge the status quo without fear of humiliation, retaliation, or punishment. In academic nursing, psychological safety is key for fostering courageous teaching, honest dialogue, and ethical advocacy; when leaders cultivate this safety, faculty are more likely to engage in reflective practice, admit mistakes, and pursue innovation (Beard and Johnson 2024; L. Butler and Lyman 2025; Madsgaard et al. 2022; Mrayyan and Al‐Rjoub 2024; Osmanović‐Zajić and Maksimović 2020). Within the HUMANE model, these dynamics support Moral Resilience and Nurturing Belonging, creating environments where compassion and honesty coexist, and where moral distress is addressed with organizational empathy rather than silence. Moral resilience, as used in this paper, refers to the capacity of individuals and collectives to sustain or restore moral integrity in the face of ethical adversity, institutional constraint, and persistent exposure to conditions that threaten core professional values (Rushton et al. 2021; Hughes and Rushton 2022). It is not synonymous with stoicism or endurance. Rather, moral resilience encompasses (a) moral awareness: the ability to recognize ethical dimensions of practice; (b) moral agency: the capacity to voice concerns and take ethically grounded action; (c) moral courage: the willingness to act despite risk; and (d) relational and organizational supports that enable these capacities to be exercised (Rushton et al. 2021; Stephens and Layne 2023). Within HUMANE, moral resilience is therefore understood as a relational and structural capacity, cultivated through psychological safety, ethical leadership, participatory governance, and cultures of belonging, rather than as an individual responsibility alone.

ESG principles, grounded in global standards for ethical and sustainable practices, provide an essential framework for transforming nursing academia into an accountable, justice‐driven institution (Sueyoshi and Goto 2025; Verna and D'Andreamatteo 2025). In the context of education, the Environmental pillar extends beyond the physical environment to include psychological safety, work–life harmony, and spaces that promote reflection and care (Hammoudi Halat et al. 2023). The Social pillar encompasses metrics related to equity, inclusion, and belonging, ranging from fair promotions to representation in decision‐making and the recognition of decolonial scholarship (Bewer et al. 2025; Iheduru‐Anderson 2025b). The Governance pillar emphasizes transparency, ethical decision‐making, and participatory leadership structures that ensure accountability for the well‐being of faculty and students (National Academies of Sciences et al. 2021; Owen et al. 2018). These aspects align with global nursing priorities, which emphasize safe, equitable, and empowering workplaces as essential for maintaining a sustainable nursing workforce (ICN 2025; WHO 2021). Within HUMANE, ESG principles are most evident in Accountability and Upholding Rights, reframing faculty well‐being as a collective indicator of institutional justice and organizational integrity. In this view, ethical governance and social responsibility are essential to sustaining equitable, transparent, and humane academic environments, positioning faculty well‐being as a barometer of an institution's moral and structural health.

The theory of tempered radicalism offers a conceptual account of how change unfolds within deeply rooted systems (Meyerson 2003). Tempered radicals are individuals who, while strongly committed to their organizations, challenge injustice through small yet principled acts of resistance. They balance the desire to belong with the need to create change, a stance many nurse educators adopt as they confront racial bias, workload inequality, and punitive cultures (C. M. Clark and Springer 2010; Small et al. 2024). HUMANE supports and legitimizes this form of leadership by offering faculty a strategic language and a moral framework for resistance that channels isolated acts of dissent into coordinated values‐driven change grounded in compassion, accountability, and systemic vision. Empowerment in HUMANE builds on both Freire's concept of critical consciousness and organizational empowerment theory in nursing. Empowerment is more than motivation; it involves redistributing power through access to resources, information, and support, which predicts job satisfaction, creativity, and moral resilience (Baker et al. 2011; Greco et al. 2006; Kanter 1977; Laschinger et al. 2015; Sarmiento et al. 2004). Conversely, disempowerment undermines psychological safety, creativity, and moral integrity (Boamah 2022; Mrayyan and Al‐Rjoub 2024). Within HUMANE, Empowerment is not a static outcome but an ongoing collective practice that moves well‐being beyond personal recovery to institutional transformation, demanding that universities dismantle exploitative workloads, democratize governance, and recognize contributions to justice and belonging.

Human rights–based frameworks further elevate faculty well‐being as a matter of global ethics. Human rights–based frameworks conceptualize well‐being as grounded in inherent human dignity and institutional obligation, rather than as a discretionary benefit or an individual responsibility. Grounded in the principle that all people possess equal and inalienable rights, these frameworks emphasize that institutions have affirmative duties to respect, protect, and fulfill conditions necessary for individuals to live and work with dignity, safety, and moral agency (Assembly, United Nations 1949; Yamin 2015). Applied to nursing education, a rights‐based orientation positions fair working conditions, freedom from discrimination, psychological safety, and the ability to exercise ethical judgment as fundamental professional rights rather than optional supports. The Universal Declaration of Human Rights affirms fair working conditions and the highest attainable standard of health (Assembly, United Nations 1949). The ICN Code of Ethics acknowledges nurses' rights to moral agency and safe environments (ICN 2021). At the same time, the ICN/WHO *State of the World's Nursing 2025* report highlights decent work, equitable education, and supportive professional environments as prerequisites for advancing global health equity (ICN 2025; WHO 2021). These commitments illustrate how HUMANE links the micro‐level (i.e., faculty health, lived experience, and moral agency) to the meso‐level (i.e., departmental culture, governance, and workload systems) and the macro‐level (i.e., workforce sustainability, human dignity, and global nursing capacity). Within this model, Upholding Rights translates human rights principles into institutional responsibilities; Healing calls for the acknowledgment and repair of moral injury; and Accountability ensures that harmful practices are actively disrupted through transparent, justice‐centered action.

Together, these theoretical foundations position HUMANE as a globally connected and ethically rigorous framework that restores moral integrity in nursing education by integrating liberation (Burke 1976; Freire 1978; hooks 1994), leadership (Bass and Riggio 2006; Boamah 2022; Cummings et al. 2008; Edmondson 2019), structural justice (Drafahl 2020; Hebenstreit 2012; Owen et al. 2018; Sueyoshi and Goto 2025; Verna and D'Andreamatteo 2025), and global ethics (Ballout et al. 2025; ICN 2021; Iheduru‐Anderson and Waite 2024; National Academies of Sciences et al. 2021; Roy et al. 2022; WHO 2021) into a unified vision of academic culture where healing is systemic, equity is made visible, and empowerment is collective. In this vision, nurse educators are not merely resilient survivors of broken systems but architects of humane institutions capable of modeling the justice and compassion they aim to teach.

## 4 The HUMANE Framework

### 4.1 Overview

**Figure 1:**
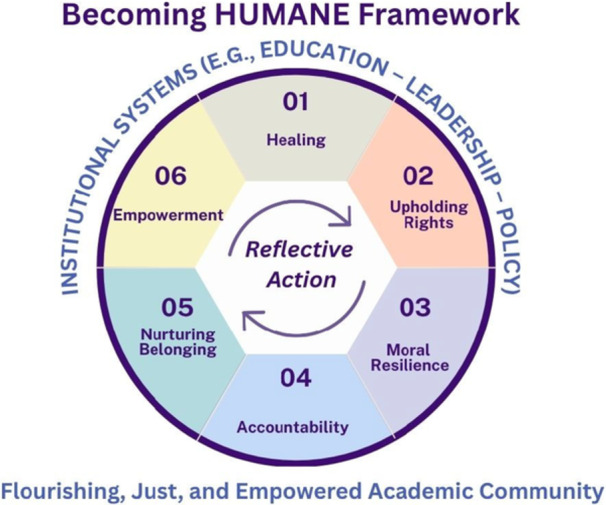
The HUMANE Framework architecture. The framework illustrates the interrelated domains of **H**ealing, **U**pholding Rights, **M**oral Resilience, **A**ccountability, **N**urturing Belonging, and **E**mpowerment, anchored in reflective action as the moral and pedagogical core. Operating within institutional systems of education, leadership, and policy, the model envisions a flourishing, just, and empowered academic community.

Figure 1 presents the HUMANE Framework as a cyclical model organized around six moral domains: Healing, Upholding Rights, Moral Resilience, Accountability, Nurturing Belonging, and Empowerment, activated by Reflective Action at its center. The HUMANE Framework is a living, relational ecosystem that positions faculty well‐being and moral integrity as the foundation and the outcome of institutional transformation. It moves beyond the static logic of wellness programs or resilience training by framing well‐being as a cyclical and reciprocal process, a continuous exchange between personal healing, moral action, and structural empowerment (National Academies of Sciences et al.; WHO 2021). Through its six interdependent domains, HUMANE serves as a regenerative moral compass, transforming individual and institutional life in nursing education.

At its center, *Reflective Action* animates the cycle, embodying the praxis of reflection and transformation described by Freire and expanded in contemporary leadership and empowerment theories (Cummings et al. 2008; Freire 1978; Kanter 1977; Sarmiento et al. 2004; Waite and Nardi 2019). Reflection without action risks moral complacency, while action without reflection reproduces institutional harm. HUMANE bridges this divide by inviting faculty and leaders to engage in intentional cycles of awareness, dialogue, and reform. Each domain feeds the next, where healing creates the psychological space for justice; rights ground healing in moral structure; resilience converts conviction into ethical agency; accountability translates values into systems; belonging nurtures safety and trust; and empowerment redistributes power to sustain change (Beard and Johnson 2024; Edmondson 2019; Mrayyan and Al‐Rjoub 2024). Together, they form a dynamic loop rather than a linear sequence, ensuring that growth, justice, and restoration remain continuous and shared. Healing initiates this cycle by acknowledging the invisible wounds of academic life (i.e., burnout, moral distress, and exclusion) that erode meaning and engagement. It is not only self‐care; it is the act of restoring humanity within systems that have normalized suffering (H. Butler 2023; Hosseini et al. 2022; Toescher et al. 2020). Reflective circles, trauma‐informed mentorship, and well‐being dialogues model compassionate accountability and collective repair (see Table 1 for illustrative mechanisms) (Evans‐Agnew et al. 2025). Healing fosters the moral and emotional conditions for reflection, empathy, and moral clarity, the essential first step toward empowerment. From this foundation emerges *Upholding Rights*, the moral infrastructure of HUMANE. Grounded in the ICN Code of Ethics (2021) and UN human rights principles, this domain reframes faculty well‐being as a right, not a privilege (Assembly, United Nations 1949; ICN 2021; Yamin 2015). Upholding Rights compels institutions to translate compassion into policy and to ensure equitable workloads, transparent promotions, and safe environments free from retaliation and bias (Akintade et al. 2025; Bewer et al. 2025; Iheduru‐Anderson 2025a, 2025b). Rights‐based governance transforms the invisible moral labor of faculty into an explicit ethical commitment, embedded in accreditation standards, evaluation systems, and leadership accountability.

**Table 1:** Component descriptions and actions.

Component	Definition in academic context	Illustrative practices	Theoretical anchors
H—Healing	Restorative acknowledgment of trauma and moral distress.	Reflective circles, well‐being debriefs, trauma‐informed mentorship.	Trauma‐informed care; oppression theory.
U—Upholding Rights	Institutionalizing dignity and equity in policy.	Faculty Rights Charter; transparent promotion criteria.	Human rights; ESG social pillar.
M—Moral Resilience	Sustaining ethical integrity amid systemic strain.	Ethics rounds, values‐based dialogue, reflection time.	Moral resilience theory; transformational leadership.
A—Accountability	Leadership and system responsibility for well‐being.	Equity dashboards; annual DEI impact reports.	ESG governance; ICN ethics.
N—Nurturing Belonging	Psychological safety and inclusive collegial community.	Mentorship networks; recognition of diverse scholarship.	Social belonging theory; psychological safety.
E—Empowerment	Collective agency and voice in decision‐making.	Shared governance councils; faculty leadership incubators.	Freirean pedagogy; tempered radicalism; transformational leadership.

*Moral Resilience*, as used in this paper, refers to the capacity of individuals and collectives to sustain or restore moral integrity in the face of moral adversity, ethical uncertainty, and institutional constraint (Rushton et al. 2021; Hughes and Rushton 2022). It encompasses core capacities including moral awareness (i.e., recognizing ethical dimensions of practice), moral agency (i.e., the ability to voice concerns and take ethically grounded action), moral courage (i.e., acting in accordance with values despite risk), and relational and organizational supports that enable these capacities to be exercised (Rushton et al. 2021; Stephens and Layne 2023). Moral resilience connects the internal moral landscape of faculty to the external pressures of institutional life; it reflects the courage to uphold ethical integrity amid competing demands and systemic strain. Within HUMANE, moral resilience is fortified not through endurance of harmful conditions but through hardiness, collective reflection, ethical dialogue, and shared decision‐making practices that strengthen agency while resisting injustice. When nurtured through psychological safety (Edmondson 2019) in a context of transformational leadership (Cummings et al. 2008), moral resilience becomes a community capacity rather than an individual burden. Relational integrity is central to this understanding of moral resilience. It refers to the preservation of ethical coherence, trust, and mutual accountability within relationships, such that individuals are not required to fragment their values to belong or succeed (Rushton et al. 2021; Hughes and Rushton 2022). HUMANE operationalizes relational integrity through its intertwined domains of Nurturing Belonging and Accountability: creating environments where faculty can speak truthfully, experience recognition, and expect consistent, values‐aligned responses from leadership (Beard and Johnson 2024; L. Butler and Lyman 2025; Owen et al. 2018). In this way, moral resilience and HUMANE are synergistic: relational integrity emerges from humane institutional conditions and serves as a mechanism through which those conditions are sustained. It empowers educators to voice concerns, challenge injustice, and model moral courage for their students (Mrayyan and Al‐Rjoub 2024). In doing so, moral resilience is sustained through relational integrity across relationships and institutional life (Rushton et al. 2021; Hughes and Rushton 2022). *Accountability* grounds these ideals in structure and transparency. In HUMANE, it represents the organizational commitment to ensure that justice and well‐being are measurable, resourced, and continuously evaluated. This domain is expressed through shared governance councils, ESG‐aligned reporting, and institutional dashboards that track equity, safety, and well‐being (Table 1) (Chen et al. 2024; Owen et al. 2018; Sueyoshi and Goto 2025; Verna and D'Andreamatteo 2025). Accountability transforms moral aspirations into systems of responsibility. It ensures that healing is sustained by policy, not sentiment, and that resilience is reinforced through ethical, transparent leadership and participatory governance. The next domain, *Nurturing Belonging*, represents the social and relational fabric of the framework. Belonging is the antidote to alienation as it transforms accountability from compliance into care (Anderson 2024; C. M. Clark et al. 2024; Dingle 2025). Within HUMANE, belonging is not treated as an interpersonal nicety but as a structural condition of ethical work. It centers on psychological safety, inclusion, and relational trust as prerequisites for innovation and ethical growth (Beard and Johnson 2024; L. Butler and Lyman 2025). When educators feel seen, supported, and valued, they are more likely to extend those same conditions to students and colleagues. HUMANE redefines belonging as a structural commitment, not an emotional extra: it requires equitable recognition, mentorship, and redistribution of institutional voice and power (Akintade et al. 2025). Through belonging, the individual and collective heal together.

Finally, Empowerment completes and regenerates the cycle. Drawing on Freire's critical pedagogy and Kanter's empowerment theory, this domain transcends personal agency to foster collective transformation (Freire 1978; Kanter 1977; Laschinger et al. 2015). Empowerment in HUMANE means that faculty not only survive but also shape the institutions they inhabit. It democratizes decision‐making, amplifies the voices of marginalized individuals, and ensures that the power to define values, policies, and curricula is shared equitably (Baker et al. 2011; Greco et al. 2006). Empowerment transforms healing into praxis: the moment when reflection becomes reform and justice becomes the lived culture of the institution. Each turn of the HUMANE cycle deepens institutional consciousness. As healing fosters courage, rights reinforce justice, and belonging anchors safety, empowerment renews the entire ecosystem, creating conditions for sustained flourishing. The framework's cyclical nature underscores that no phase is ever complete; institutions and individuals can continually revisit, realign, and re‐engage. This regenerative process mirrors nursing itself as a profession of ongoing care, reflection, and renewal (Bewer et al. 2025; C. M. Clark et al. 2024; Roy et al. 2022). HUMANE therefore embodies nursing's moral promise: to heal self and system together. The cyclical design of HUMANE operationalizes tempered radicalism (Meyerson 2003), offering faculty an analytic guide for leading change within constraints. By cultivating reflection, community, and shared purpose, HUMANE transforms moral distress into moral imagination, an active stance that transforms fatigue into creativity, isolation into solidarity, and despair into collective action. When HUMANE is treated as an ongoing institutional practice rather than a discrete program, institutions can move beyond reactive approaches to burnout toward moral ecosystems grounded in care, accountability, and ethical coherence, where individual healing becomes the seed of institutional empowerment. Empowerment sustains the soil for continuous healing, inviting nursing education to become what it teaches: humane.

## 5 Framework Distinctiveness

Across higher education, numerous frameworks have emerged to support faculty and staff well‐being, ranging from the American College Health Association's Healthy Campus Framework to system‐level approaches to faculty flourishing and psychological safety (American College Health Association [ACHA] 2021, 2025; Pope‐Ruark 2024). These models indicate a growing awareness that institutional health is closely linked to educators' well‐being. However, most frameworks still focus on individual coping, productivity, and organizational efficiency, reflecting managerial rather than moral or justice‐based perspectives (P. M. Johnson 2022; R. Johnson 2022). Within this landscape, nursing education presents a distinctive challenge. Faculty in schools and colleges of nursing operate at the intersection of professional care ethics and academic labor systems. They are expected to model compassion, moral resilience, and trauma‐informed leadership for students, even as they navigate institutional inequities, racialized hierarchies, and escalating workload pressures (Akintade et al. 2025; Ballout et al. 2025; Iheduru‐Anderson et al. 2025; Waite and Nardi 2019). Despite widespread awareness of faculty burnout and moral distress, few frameworks explicitly link nursing's ethical obligations to the rights and health of those who teach it (Hughes and Rushton 2022; Rushton et al. 2021, 2022). The HUMANE Framework addresses this gap by re‐centering nursing faculty as the primary site of care, reflection, and institutional renewal. Yet despite the abundance of well‐being frameworks in higher education, few directly address the moral, relational, and justice‐centered dimensions of faculty well‐being that are intrinsic to nursing's professional identity.

Unlike generalized higher education models, such as the Healthy Campus Framework (ACHA 2021), which promote organizational wellness through strategic planning and health promotion activities, HUMANE positions faculty well‐being as a moral and human rights imperative, asserting that educators' mental, emotional, and moral health form the ethical foundation of academic integrity, student flourishing, and social accountability (Assembly, United Nations 1949; ICN 2021). Whereas higher education wellness models emphasize culture change through infrastructure and programming, HUMANE redefines culture as a living moral ecosystem, sustained through reflection, rights‐based governance, and collective empowerment (Burke 1976; Freire 1978; hooks 1994). This distinction is also clear when compared to workplace well‐being frameworks, such as the U.S. Surgeon General's Five Essentials for Workplace Mental Health and Well‐being: protection from harm, connection, work–life balance, mattering, and growth (Office of the Surgeon General 2024). HUMANE's cyclical structure also distinguishes it from linear wellness models by recognizing that healing, justice, and empowerment need to be continuously revisited rather than achieved once. While these principles are helpful, they are mostly descriptive and lack the decolonial, justice‐focused perspective needed to address structural inequities within nursing academia. HUMANE interprets these same essentials through a moral lens: Healing restores wholeness from harm, Belonging fosters genuine connection, Empowerment gives voice and agency, and Accountability promotes institutional transparency (Edmondson 2019; Rasche 2020). In doing so, HUMANE shifts well‐being from a managerial issue to a form of ethical and social repair.

Similarly, frameworks such as the AACN Healthy Work Environments Standards (American Association of Colleges of Nursing [AACN] 2015; Mabona et al. 2022) and the Magnet Model (Magnet Recognition Program®|ANCC 2017) have profoundly influenced nursing leadership and professional practice. Yet both models primarily measure excellence through patient outcomes, engagement, and retention rather than through the ethical flourishing of faculty and staff themselves. HUMANE reinterprets these standards through a rights‐based and decolonial lens, extending the definition of “health” beyond organizational success to encompass human dignity, justice, and moral sustainability (Ballout et al. 2025; Ballout and Hamadeh 2025; Iheduru‐Anderson and Waite 2024). The HUMANE Framework complements the United Nations Sustainable Development Goals 3 (ensure healthy lives and promote well‐being and Goal 10 reduce inequalities (El‐Jardali et al. 2018; Sueyoshi and Goto 2025). It invites institutions to measure excellence not by prestige or performance, but by their capacity to protect and uplift the people who constitute their moral core. From a higher education systems perspective, HUMANE also extends recent faculty well‐being frameworks that emphasize relationality and systems thinking, such as the Positive and Relational Academic Well‐Being Framework (Donaldson et al. 2022; Kucirka and Baumberger‐Henry 2021; Mixer et al. 2013; Pope‐Ruark 2024; Zwane 2025) and the systems approach to staff well‐being (Johnson 2022; Leffler and Godfrey 2025; Yadav 2025; Zhang et al. 2025). While these frameworks highlight collegiality and institutional supports, they often overlook nursing's distinctive epistemology of care and its history of gendered, racialized labor. HUMANE bridges this gap by combining Freirean and Fanonian liberation theory with ESG principles and the ICN Code of Ethics (2021), reframing faculty care as both an ethical obligation and a governance priority. It is the first framework to integrate moral resilience, decolonial healing, and human rights into a cohesive, cyclical model explicitly designed for nursing education institutions. A comparative summary of existing higher education and nursing well‐being frameworks is presented in Table 2.

**Table 2:** Comparison of selected well‐being frameworks and the becoming HUMANE Framework.

Framework	Primary focus	Level of application	Conceptual orientation	Limitations/Gaps	Distinctive contributions of HUMANE
AACN Healthy Work Environments Standards	Communication, collaboration, leadership, staffing, recognition	Clinical and academic nursing settings	Professional standards; performance‐ and safety‐based	Focuses on work conditions and performance, not moral or human rights dimensions	Reinterprets “health” as moral and justice‐based; centers faculty well‐being and structural accountability
Magnet Model	Transformational leadership, structural empowerment, exemplary practice	Healthcare institutions and nursing excellence programs	Organizational performance and quality improvement	Measures excellence through patient outcomes and engagement; limited attention to faculty well‐being	Expands excellence to include faculty flourishing, belonging, and empowerment
Healthy Campus Framework	Institutional health promotion and culture change	Higher education institutions	Systems‐based, health‐promoting campus model	Focuses broadly on physical and mental health infrastructure; lacks ethical grounding	Frames well‐being as a moral right and governance obligation within nursing education
U.S. Surgeon General's Framework for Workplace Mental Health	Protection from harm, connection, work–life balance, mattering, growth	General workforce and higher education contexts	Psychological and behavioral health model	Descriptive rather than transformative; lacks decolonial and rights‐based approach	Interprets essentials through moral domains: Healing, Belonging, Empowerment, and Accountability
Positive and Relational Academic Well‐Being Frameworks	Collegiality, relational well‐being, systems supports	Higher education institutions	Positive psychology and relational constructionism	Limited to relational or interpersonal interventions; omits structural inequities	Integrates relational, decolonial, and structural empowerment approaches for systemic change
Becoming HUMANE Framework	Healing, Upholding Rights, Moral Resilience, Accountability, Nurturing Belonging, Empowerment	Nursing education institutions	Rights‐based, decolonial, tempered‐radical model	—	Positions faculty as the first site of care; unites healing and activism through reflective action; links personal and institutional empowerment

What ultimately distinguishes HUMANE is its tempered radicalism and its insistence that transformation begins within existing institutions, rather than outside them. Rather than positioning well‐being as an endpoint, HUMANE frames it as a regenerative process that links individual healing with institutional empowerment. Nursing faculty are recast not as passive recipients of policy but as agents of moral and structural change (Ballout and Hamadeh 2025; Cummings et al. 2008; Edmondson 2019; Iheduru‐Anderson et al. 2025; Waite and Nardi 2019). Through reflective action and mutual accountability, they model the humanity they teach, demonstrating how systemic care begins with self and extends outward to the organizational and societal transformation. In essence, HUMANE reframes the question from “How can we help faculty cope?” to “How can institutions become humane?” By positioning healing, justice, and belonging as structural commitments, HUMANE transforms the ideal of a “healthy work environment” into a vision of a flourishing, just, and empowered academic community.

## 6 Institutionalizing HUMANE in Nursing Education

The HUMANE Framework offers an analytic orientation for translating moral commitments into institutional conditions that link individual healing with systemic responsibility, rather than treating well‐being as an isolated wellness initiative. Table 3 summarizes illustrative alignments across faculty development, leadership, curriculum, and governance.

**Table 3:** Operationalizing the HUMANE Framework in nursing education.

Institutional domain	Primary mechanism	Operational strategies	HUMANE domains activated
Faculty development	Moral and restorative reflection	Ethics circles; restorative dialogues; self‐advocacy workshops; trauma‐informed mentoring	Healing, empowerment, belonging
Leadership Implementation	Transformational mentorship and ESG integration	Distributed leadership structures; well‐being metrics in ESG reports; reflective supervision; shared accountability councils	Moral resilience, accountability
Curriculum design	Rights‐based pedagogy and experiential learning	Embedding equity and advocacy modules; Freirean dialogue; community‐engaged projects; reflective journals	Rights, empowerment, healing
Policy and governance	Structural embedding of equity and care	Faculty well‐being standards; justice impact assessments; transparent reporting and evaluation	Accountability, rights, empowerment

### 6.1 Faculty Development: From Reflection to Renewal

A HUMANE perspective reframes faculty development as collective moral repair and renewal, not merely productivity, pedagogy, or scholarly output. This orientation also recognizes that such repair and renewal cannot be presumed; rather, faculty development must intentionally cultivate moral agency, moral integrity, and moral efficacy, supporting educators in clarifying the ethical contours of their role and strengthening their capacity to recognize, articulate, and act upon moral concerns (Rushton et al. 2021; Hughes and Rushton 2022; Stephens and Layne 2023). As summarized in Table 3, this includes institutional supports that normalize ethical reflection, repair, and rights‐based recognition of invisible labor. Consistent with scholarship on ethical practice environments, such supports extend beyond discrete programs to encompass organizational conditions that make ethical dialogue routine, make moral concerns speakable, and enable repair following harm (Rushton et al. 2021; Hughes and Rushton 2022). Ethical practice environments are characterized by transparent decision‐making, participatory governance, leadership accountability, and access to resources that enable individuals and groups to act in alignment with their values. Within HUMANE, these conditions provide the infrastructural scaffolding through which moral resilience, relational integrity, and moral agency can be collectively cultivated rather than left to individual effort. In traditional models, professional growth is usually evaluated based on scholarly output or teaching innovation. A HUMANE‐aligned approach redefines development as collective renewal, creating spaces where educators can reflect, heal, and regain their sense of control. Restorative dialogue circles and ethics reflection groups provide opportunities for moral healing, moral agency, and relational integrity, as core capacities of moral resilience, enabling educators to process ethical challenges, reclaim voice, and re‐engage with their professional values (Edmondson 2019; Stephens and Clark 2024). They also support the Healing and Belonging domains by framing vulnerability as a sign of moral integrity rather than weakness (Evans‐Agnew et al. 2025; Rushton et al. 2021, 2022). Self‐advocacy workshops extend this restoration into empowerment, teaching faculty to navigate structural inequities and advocate for fair evaluation, workload balance, and recognition of invisible labor (Akintade et al. 2025; Iheduru‐Anderson 2025b).

### 6.2 Leadership Implementation: Modeling Moral Courage and Accountability

A HUMANE lens implies that leadership is ethically consequential not only through relational style but through how power is exercised, whether it protects voice, legitimizes dissent, and sustains accountability for harm. This ethical orientation cannot be presumed; rather, HUMANE underscores the need for leadership development that intentionally cultivates moral awareness, moral agency, relational integrity, and the skills required to engage ethical tension, hold power accountably, and respond to harm (Hughes and Rushton 2022; Rushton et al. 2021; Edmondson 2019; Owen et al. 2018). Table 3 provides examples of how these conditions can be institutionalized through participatory governance and transparent accountability structures. Leaders serve not as gatekeepers but as facilitators of ethical consistency while aligning the institution's mission with the lived culture. Transformational mentorship programs train chairs, deans, and emerging leaders to lead with humility, courage, and transparency (Cummings et al. 2008; Mrayyan and Al‐Rjoub 2024). This form of leadership models Moral Resilience and Accountability, creating conditions where open dialogue and dissent are recognized as forms of institutional loyalty rather than defiance, by institutionalizing transparent decision‐making, protecting faculty from retaliation when raising concerns, and creating routine, psychologically safe spaces for ethical dialogue and collective problem‐solving (Edmondson 2019; Hughes and Rushton 2022; Meyerson 2003; Owen et al. 2018; Rushton et al. 2021). Leaders cultivate HUMANE's moral architecture by creating environments where faculty can voice ethical concerns without fear of reprisal. Non‐punitive responses to errors and trauma‐informed practices model institutional courage and reinforce psychological safety, enabling faculty to challenge injustice and advocate for ethical action (Hughes and Rushton 2022). In such a climate, policies reflect values like compassion and justice, while ethical, inclusive leadership and values‐based decision‐making are recognized. System‐level tools are therefore essential. Systematic operationalization of these values is supported by scholarship on ethical practice environments, which emphasizes participatory governance, transparent decision‐making, routinized ethical dialogue, and continuous organizational learning as core infrastructures for sustaining ethical climates (Rushton et al. 2021; Hughes and Rushton 2022; Owen et al. 2018). Psychological safety further functions as a foundational process condition, enabling individuals to surface concerns, question assumptions, and participate meaningfully in institutional improvement (Edmondson 2019). National guidance similarly emphasizes the importance of embedding ethics, equity, and well‐being into policies, evaluation frameworks, and leadership accountability structures rather than relying on discretionary or episodic initiatives (National Academies of Sciences et al. 2021). Embedding ESG principles into reporting and performance systems further reinforces HUMANE's structural dimension. When faculty well‐being, belonging, and equity are treated as governance indicators, alongside fiscal or research metrics, they become part of the institution's ethical infrastructure (Sueyoshi and Goto 2025; Verna and D'Andreamatteo 2025).

### 6.3 Curriculum Design: Teaching Equity as Praxis

HUMANE suggests that curriculum is not only a site of content delivery but a moral site where institutions either reproduce or interrupt domination, shaping whether equity is treated as praxis rather than rhetoric. Examples of curricular alignment with reflective action and rights‐based ethics are summarized in Table 3. HUMANE further emphasizes the intentional cultivation of moral resilience competencies within student formation, including moral awareness, moral agency, moral courage, and moral integrity, not as a means of tolerating unethical conditions but as preparation to recognize injustice, voice concern, and act in alignment with professional and ethical obligations (Rushton et al. 2021; Hughes and Rushton 2022; Stephens and Layne 2023). Integrating HUMANE principles into course design, pedagogy, and learning assessment ensures that the culture of healing and justice permeates the entire educational experience. A rights‐based and empowerment‐oriented curriculum integrates global ethical frameworks (i.e., ICN, WHO, and UN Global Compact) into nursing courses, framing equity as a fundamental professional competency (Assembly, United Nations 1949; ICN 2025; WHO 2021). Freirean dialogue, community engagement, and reflective journaling are used to cultivate critical awareness of power, oppression, and advocacy (Burke 1976; Freire 1978; Waite and Nardi 2019). The READY RISE Incubator serves as a case illustration (Ballout et al. 2025). Designed to foster equity‐centered leadership, it integrates modules on reflective action, systems thinking, and anti‐oppressive practice (Astle 2021; Ballout 2025a; Berthoud 2024). Students and faculty engage in collaborative inquiry that links personal growth with institutional transformation. In this setting, learning itself becomes a healing and empowering act, aligning with the HUMANE vision of moral and psychological safety as preconditions for excellence (Ballout and Hamadeh 2025; H. Butler 2023; Chun and Evans 2023; Chung and Kowalski 2012; Edmondson 2019; Greco et al. 2006; Mrayyan 2024). Integrating HUMANE principles into curricula reinforces the framework's cyclical nature: reflection fosters insight, deepening agency and leading to structural transformation.

### 6.4 Policy and Governance: Institutionalizing Care and Justice

HUMANE reframes governance as the central mechanism through which institutions either individualize distress or assume ethical responsibility for the conditions that produce moral injury. Table 3 outlines illustrative governance mechanisms that shift care from aspiration to accountability (e.g., equity and well‐being indicators, workload justice, and transparent promotion practices). Institutions often depend on short‐term wellness programs or diversity pledges that lack accountability measures (ICN 2025; National Academies of Sciences et al. 2021; WHO 2021). HUMANE provides a framework for embedding care, equity, and ethical responsibility directly into an organization's core. Accreditation and assessment processes can include well‐being standards that emphasize relational leadership, inclusive mentorship, and moral resilience as signs of professional excellence (AACN 2015; ACHA 2021, 2025; Iheduru‐Anderson et al. 2023; Magnet Recognition Program®|ANCC 2017; Yadav 2025). Faculty performance evaluations can incorporate justice impact assessments, fostering reflective evaluation of teaching and service practices that promote belonging and rights.

By shifting from resilience‐as‐endurance to resistance‐as‐ethical practice, HUMANE invites a different question: What institutional arrangements would need to change for faculty to sustain moral integrity without sacrificing their health or humanity? Rushton's model of moral resilience offers an implementation‐oriented roadmap for addressing this question by pairing the cultivation of moral‐resilience capacities with deliberate system design to create ethical practice environments, shifting responsibility from individual endurance toward organizational accountability and structural conditions that enable integrity (Rushton 2024; Rushton and Sharma 2018). As defined earlier, we draw on established scholarship on moral resilience, moral injury, moral courage, moral imagination, and moral integrity. We use these concepts collectively to describe the moral terrain nurse educators navigate.

## 7 Discussion: HUMANE as a Tempered Radical Practice

The HUMANE Framework promotes a vision of nursing education as a moral pursuit and a space for systemic change. As an ethical model, it defines an ethics of care based on healing, rights, and accountability; as a conceptual lens, it provides a guide for turning those ethics into practice through reflection, policy, and governance. HUMANE encourages educators, leaders, and institutions to reimagine well‐being not just as the absence of distress but as the presence of justice, dignity, and belonging (ICN 2021; National Academies of Sciences et al. 2021). Its cyclical architecture distinguishes it from linear wellness models by emphasizing that healing, justice, and empowerment need to be continually revisited rather than achieved once. In doing so, it addresses a growing need in higher education and nursing: to humanize systems that have long relied on faculty endurance rather than institutional empathy (Hosseini et al. 2022; Pope‐Ruark 2024; Singh et al. 2020). HUMANE's strength lies in framing faculty as tempered radicals or professionals who remain committed to their institutions while exercising moral courage to challenge injustice from within (Meyerson 2003). Within nursing, HUMANE operationalizes this stance by providing structured practices, rights‐claiming mechanisms, and collective supports that translate moral tension into coordinated, values‐driven institutional change (Akintade et al. 2025; Drafahl 2020; Small et al. 2024; Stephens and Layne 2023). HUMANE renders tempered radicalism within institutional life by providing structured practices, reflection, rights‐claiming, and collective empowerment that channel personal moral tension into systemic change. Read through this lens, tempered radicalism in nursing education cannot be separated from the political economy shaping institutional life. By incorporating the moral areas of Healing, Upholding Rights, Moral Resilience, Accountability, Nurturing Belonging, and Empowerment, HUMANE provides educators with the language and framework to maintain ethical bravery in the face of obstacles. It turns moral distress into moral imagination, a capacity to envision and create new possibilities for care within existing institutional systems (Toescher et al. 2020).

Research underscores the necessity of such moral resistance (Iheduru‐Anderson, Akanegbu, and Ugorji 2025; Iheduru‐Anderson, Akanegbu, Ugorji, et al. 2025). This work on the racialized experiences of Black nurse educators exposes how institutional whiteness, epistemic exclusion, and inequitable labor expectations generate emotional harm and moral fatigue. Faculty empowerment, in this context, becomes an act of defiance and a refusal to normalize marginalization. These studies demonstrate that moral distress among minoritized faculty is not an individual deficit but a structural outcome of institutionalized inequity, a condition HUMANE directly addresses through its Healing, Rights, and Empowerment domains. Similarly, Ballout (2025) emphasizes that decolonizing nursing education requires curricular reform and the rehumanization of academic structures themselves (Ballout et al. 2025; Ballout and Hamadeh 2025). HUMANE builds upon these insights by reframing empowerment as a collective and systemic force: healing and resistance are no longer opposites but interdependent acts of survival and transformation. When faculty practice HUMANE principles, they enact moral agency through self‐care and through care‐as‐change, refusing to reproduce harm while cultivating cultures of belonging (Anderson 2024; C. M. Clark et al. 2024; Dingle 2025; Edmondson 2019; Stephens and Clark 2024).

Within this political–economic landscape, HUMANE offers more than a framework for well‐being; it functions as a moral grammar for contesting austerity and reclaiming academic care as a governance obligation (Alexander 2025; Owen et al. 2018; Pope‐Ruark 2024; Verna and D'Andreamatteo 2025). Accountability within HUMANE requires naming how resource‐allocation decisions either reproduce or disrupt harm (Flaubert et al. 2021; Hughes and Rushton 2022; National Academies of Sciences et al. 2021; WHO 2021). Empowerment requires legitimizing faculty organizing, cross‐unit solidarity, and collective refusal to normalize disinvestment (Baker et al. 2011; Freire 1978; Greco et al. 2006; Kanter 1977; Laschinger et al. 2015). Healing acknowledges that repeated exposure to institutional abandonment, despite good‐faith advocacy, produces grief, anger, and exhaustion that are rational responses to structural violence rather than individual weakness (H. Butler 2023; Hughes and Rushton 2022; Rushton et al. 2022; Stephens and Layne 2023). In this sense, faculty advocacy that persists even when met with silence exemplifies tempered radicalism: remaining committed to the institution while simultaneously refusing its moral incoherence (C. M. Clark and Springer 2010; Meyerson 2003; Small et al. 2024). HUMANE reframes such labor not as futile idealism, but as ethically necessary resistance (Ballout et al. 2025; Iheduru‐Anderson and Waite 2024; Rushton et al. 2021). Read through this lens, HUMANE also contests regimes of manufactured urgency. By insisting that healing, reflection, and collective deliberation are ethical necessities rather than luxuries, HUMANE challenges institutional cultures that equate speed with excellence and productivity with value (Edmondson 2019; Pope‐Ruark 2024; Hughes and Rushton 2022). Accountability within HUMANE, therefore, includes responsibility for time: protecting space for reflection, relationship, and repair as core conditions of moral integrity (Flaubert et al. 2021; National Academies of Sciences et al. 2021; WHO 2021).

**Figure 2:**
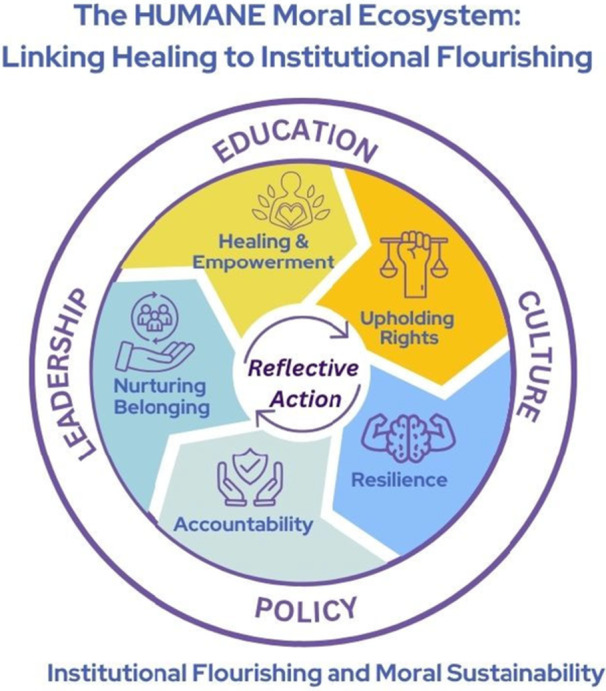
The HUMANE Moral Ecosystem: linking healing to institutional flourishing. Reflective action connects the six moral domains—Healing and Empowerment, Upholding Rights, Moral Resilience, Accountability, and Nurturing Belonging—across institutional systems of education, leadership, culture, and policy to achieve institutional flourishing and moral sustainability.

Building on earlier definitions of moral resilience, moral agency, moral integrity, and relational integrity, and emphasizing systematic rather than solely individual‐level responses, we acknowledge that, despite HUMANE's transformative potential, several structural forces constrain its implementation. Yet, HUMANE within contemporary academia is not without barriers. Structural performance metrics often reward productivity over reflection, compliance over conscience. The corporatization of higher education perpetuates burnout by prioritizing publication counts and grant revenue over mentorship, inclusion, and ethical leadership (Alexander 2025; H. Butler 2023; Evans 2007; Hata et al. 2022; Pope‐Ruark 2024; Verna and D'Andreamatteo 2025). Emotional labor, particularly for women and faculty of color, remains unacknowledged and uncompensated, creating invisible inequities that undermine well‐being (Akintade et al. 2025; Archer 2025; H. Butler 2023; Evans 2007). These conditions erode moral resilience and normalize cynicism as a coping mechanism. Without systemic change, calls for individual resilience risk perpetuate the very harm they seek to remedy. Against these forces, HUMANE identifies enablers of transformation that sustain tempered radical practice. Leadership commitment is essential when deans, chairs, and senior administrators model transparency, compassion, and moral accountability, thereby normalizing ethical discourse in decision‐making. Communities of practice, such as restorative dialogue groups, peer mentoring networks, and interfaculty equity councils, offer spaces of solidarity and healing where reflection becomes action. ESG principles provide an accountability mechanism that moves HUMANE from aspiration to sustained practice by embedding well‐being and moral climate into governance metrics. These enablers enact moral resilience as a collective capacity embedded within institutional relationships and structures, creating cultures that sustain rather than silence integrity, consistent with the model of moral resilience and ethical practice environments described earlier. Figure 2 synthesizes the HUMANE Moral Ecosystem, showing how reflective action connects the six domains across institutional levels. This visualization captures how individual healing and empowerment extend outward into systemic transformation.

Within the broader context of the Nurses' Health and Well‐Being special issue, HUMANE represents a scalable innovation for systemic well‐being. HUMANE's distinctive contribution lies in uniting moral philosophy, decolonial praxis, empowerment theory, and ESG‐aligned governance into a cyclical, relational model of institutional well‐being, something absent in existing frameworks. It bridges individual, relational, and structural levels of transformation, offering a model adaptable across disciplines and institutional types. Its cyclical structure allows for contextual tailoring, with schools of nursing prioritizing healing and belonging while universities might emphasize governance and accountability. Across all contexts, the framework operationalizes moral courage as personal discipline and institutional design. HUMANE thus embodies nursing's dual legacy, as a profession of care and a justice movement. It transforms the faculty role from one of compliance and endurance to one of reflection, resistance, and renewal. Read as a moral framework, HUMANE does more than reduce burnout; it restores moral coherence to the academic mission. It ensures that the well‐being of those who teach care becomes the benchmark of institutional integrity. In this way, HUMANE reframes well‐being as a moral and structural mandate, positioning faculty not as survivors of strained institutions but as architects of humane academic cultures. In the words of its tempered radicals, HUMANE affirms that the future of nursing education depends not on resilience alone, but on the collective courage to make our institutions as humane as the care we teach.

## 8 Implications

The HUMANE Framework offers an integrated pathway for advancing moral resilience, equity, and institutional well‐being across the domains of practice, education, policy, and research. By articulating how healing, rights, accountability, belonging, and empowerment function as institutional conditions, HUMANE provides an analytic foundation for transforming the culture and structure of nursing education.

At the institutional level, HUMANE reframes curriculum as a moral site where commitments to healing, empowerment, and justice are either enacted or undermined. The framework implies that leadership is not value‐neutral: decision‐making, mentorship, and evaluation practices actively shape the moral climate and faculty well‐being. Reflective and restorative leadership practices, particularly those grounded in ethical reflection, trauma‐informed communication, and distributed governance, emerge as institutional conditions that foster trust and shared accountability, rendering moral integrity a core competency rather than an aspirational value.

In nursing education, integrating HUMANE across curricula deepens the moral and human rights dimensions of professional formation. Embedding content on healing, empowerment, and justice prepares graduates to navigate complex ethical landscapes while maintaining compassion for themselves and others. This pedagogical approach aligns with global nursing frameworks that emphasize care, equity, and sustainability as essential learning outcomes. It further emphasizes belonging as a structural condition (i.e., psychological safety, cultural affirmation, relational trust, and equitable support) that enables students and educators to thrive.

At the policy level, HUMANE foregrounds accreditation and regulation as sites of moral governance. By treating faculty well‐being, moral climate, and equity as indicators of educational quality, accrediting bodies implicitly shape institutional norms and incentives. These indicators can institutionalize accountability for psychological safety and inclusivity, ensuring that faculty health is recognized as a determinant of educational quality and workforce sustainability. Integrating HUMANE metrics into accreditation reviews would also support national workforce goals by reducing burnout‐driven attrition among nursing faculty.

Finally, HUMANE raises critical questions for future research, including the development of validated assessment tools, the measurement of institutional moral climate, and the examination of effects across diverse academic settings. Longitudinal and participatory studies can capture how implementing HUMANE affects faculty retention, resilience, and student outcomes, thereby building a research‐to‐practice bridge for systemic transformation. These implications and recommendations for operationalizing the Becoming HUMANE Framework across practice, education, policy, and research domains are summarized in Table 4.

**Table 4:** Implications and recommendations for operationalizing the becoming HUMANE Framework in nursing education.

Domain	Implications/Recommendations
Practice & Leadership	Adopt HUMANE principles in faculty governance, mentorship, and leadership training to promote ethical accountability, reflective practice, and inclusive decision‐making, and the cultivation of moral resilience through psychological safety, ethical dialogue, and relational integrity.
Education	Integrate content on rights, healing, empowerment, and belonging, and moral resilience competencies (moral agency, moral courage, moral imagination, and integrity) into nursing curricula to prepare graduates for ethical, equity‐centered practice.
Policy	Embed faculty well‐being and equity metrics into accreditation and licensure standards to institutionalize moral and psychological safety as quality benchmarks.
Research	Develop, validate, and implement HUMANE‐based and moral resilience–informed assessment tools and interventions across institutions to measure and strengthen academic moral climate.

## 9 Conclusion

The HUMANE Framework emphasizes that humane care cannot be taught in inhumane institutions. Nursing education, rooted in compassion, equity, and healing, often occurs within systems that devalue educators, affecting their well‐being, moral integrity, and retention. HUMANE restores the moral core of nursing education, viewing faculty well‐being as an ethical, collective responsibility, transforming moral ideals into practice through six domains. It envisions academic environments with mutual care, measurable integrity, and embedded justice, where healing and empowerment are acts of solidarity rather than self‐preservation. Supporting faculty allows them to teach and lead with wholeness, exemplifying nursing's humanity. HUMANE ultimately asks whether nursing education is willing to hold itself to the same ethical standards it teaches, treating faculty well‐being not as a benefit or buffer, but as a defining measure of institutional integrity.

## Funding

The authors received no specific funding for this work.

## Ethics Statement

This project was undertaken as a conceptual initiative and did not require IRB approval.

## Conflicts of Interest

The authors declare no conflicts of interest.

## Data Availability

Data sharing is not applicable to this article as no datasets were generated or analyzed during the current study.
